# An injectable hyperthermic nanofiber mesh with switchable drug release to stimulate chemotherapy potency

**DOI:** 10.3389/fbioe.2022.1046147

**Published:** 2022-11-03

**Authors:** Lili Chen, Nanami Fujisawa, Masato Takanohashi, Mitsuhiro Ebara

**Affiliations:** ^1^ Research Center for Functional Materials, National Institute for Materials Science (NIMS), Tsukuba, Japan; ^2^ Graduate School of Pure and Applied Sciences, University of Tsukuba, Tsukuba, Japan; ^3^ Department of Materials Science and Technology, Tokyo University of Science, Tokyo, Japan

**Keywords:** hyperthermia, injectable nanofiber, chemotherapy, magnetic nanoparticles, temperature-responsive polymers

## Abstract

We developed a smart nanofiber mesh (SNM) with anticancer abilities as well as injectability and fast recovery from irregular to non-compressible shapes. The mesh can be injected at the tumor site to modulate and control anticancer effects by loading the chemotherapeutic drug, paclitaxel (PTX), as well as magnetic nanoparticles (MNPs). The storage modulus of the mesh decreases when applied with a certain shear strain, and the mesh can pass through a 14-gauge needle. Moreover, the fibrous morphology is maintained even after injection. In heat-generation measurements, the mesh achieved an effective temperature of mild hyperthermia (41–43°C) within 5 min of exposure to alternating magnetic field (AMF) irradiation. An electrospinning method was employed to fabricate the mesh using a copolymer of *N*-isopropylacrylamide (NIPAAm) and N-hydroxyethyl acrylamide (HMAAm), whose phase transition temperature was adjusted to a mildly hyperthermic temperature range. Pplyvinyl alcohol (PVA) was also incorporated to add shear-thinning property to the interactions between polymer chains derived from hydrogen bonding, The “on-off” switchable release of PTX from the mesh was detected by the drug release test. Approximately 73% of loaded PTX was released from the mesh after eight cycles, whereas only a tiny amount of PTX was released during the cooling phase. Furthermore, hyperthermia combined with chemotherapy after exposure to an AMF showed significantly reduced cancer cell survival compared to the control group. Subsequent investigations have proven that a new injectable local hyperthermia chemotherapy platform could be developed for cancer treatment using this SNM.

## Introduction

Drug delivery systems (DDSs) are being widely investigated as technologies to reduce the effects of drugs on normal cells while specifically targeting cancer cells (Parisi et al., 2014; Sanadgol and Wackerlig, 2020). In a DDS, the drug distribution and concentration are controlled spatiotemporally to reduce side effects and improve drug efficacy by administering only the necessary and sufficient amount of the drug. In the study of DDSs in the treatment of cancer, the enhanced permeation and retention (EPR) effect discovered by Maeda et al. has often been applied (Maeda, 2010). The EPR effect refers to the fact that neovascularization, which occurs rapidly around cancerous tumors, has an incomplete structure with gaps between the vascular endothelial cells. By leaking into the interstitial tissues, nanoparticles with diameters of 100 nm or less are easily delivered to the tumor sites. A significant benefit of this effect is that it allows selective drug delivery to cancerous tumors (Maeda and Islam, 2020; Subhan et al., 2021). However, due to the uncontrollable nature of passive targeting, there are still some challenges to making EPR effect in clinical application.

The concept of a reservoir formulation has attracted attention as a locally controllable DDS from a different perspective than the EPR effect. A reservoir formulation is a drug-loaded formulation that is implanted in the patient’s body so that the drug can be released into the body at any time. The formulation is implanted under the skin or in an affected area of the patient’s body through surgery involving incision and removal (Sapino et al., 2019; Fayzullin et al., 2021); the aim here is to administer the necessary and sufficient amount of the drug at the appropriate time. The drug reservoir is often located near the affected area, allowing for physical space and temporal control through the timing of drug administration from the reservoir. Reservoir-based formulations have been used for patients who require long-term care and continuous drug administration, such as those with hormonal imbalances or osteoporosis (Villarruel Mendoza et al., 2020), patients undergoing chemotherapy supported by subcutaneous port implantation, and diabetic patients (Anselmo and Mitragotri, 2014; Sanjay et al., 2018; Li W et al., 2022) using insulin pump therapy. From these clinical experiences, it is clear that reservoir-based formulations can improve the quality of life by preventing missed or lost medications and reducing the physical and mental burden of patients due to long-term drug dependency. Therefore, we developed and studied a smart nanofiber mesh (SNM) as a new cancer treatment platform that enables active targeting of cancerous tumors by focusing on the concept of reservoir-based formulation delivery.

SNM is a type of nanofiber with “smart” properties, based on their physical or chemical properties such as “stimuli-responsive” and “environmental-sensitive”, which for such applications as “on-off” switchable control of swelling/deswelling and adhesion behavior (Wang et al., 2019; Wang et al., 2022). This material can be produced by electrospinning through a single needle (Khodadadi et al., 2020), coaxial electrospinning (Peng et al., 2021; Li J et al., 2022), or bubble spinning (He et al., 2022; Qian and He, 2022). The SNM is surgically implanted near the affected area and can effectively treat diseases through active targeting and long-term release of encapsulated drugs. As an example, an SNM targeted at carpal tunnel syndrome encapsulates a conventional therapeutic drug for carpal tunnel syndrome in a nanofiber mesh composed of polycaprolactone. The SNM has been successfully applied to stimulate the regeneration of peripheral nerves, which is not possible with a single therapeutic drug and is currently under clinical trials in humans (Grinsell and Keating, 2014; Hoyng et al., 2015).

In addition, a SNM for cancer therapy was developed using poly (*N*-isopropylacrylamide-co-N-hydroxymethyl acrylamide) (P(NIPAAm-*co*-HMAAm)), a temperature-responsive polymer, as the substrate and magnetic nanoparticles (MNPs) as the heating elements. HMAAm was used to provide the chemical crosslinking points as well as to adjust the phase transition temperature around hyperthermic temperature. The SNM was designed to induce phase transition of P(NIPAAm-co-HMAAm) by heat generation from the MNPs, thus enabling accelerated release of the drug. Furthermore, by maintaining the heat generation temperature around 42–43°C, we were able to combine hyperthermia, a kind of heat-based cancer therapy, with chemotherapy by releasing the encapsulated drug. We aim to develop a minimally invasive therapy with fewer side effects on normal cells through localized combined hyperthermia/chemotherapy.

As a method of providing injectability, we considered using the shear-thinning property in the SNM. The shear-thinning property is a phenomenon by which a material becomes fluid under an applied shear stress. This is known to occur when the shear stress increases the mobility of the molecules by breaking the intermolecular interactions, orienting molecules along the stress direction, and removing entanglements between molecules (Dufresne and Castaño, 2017; Sadasivuni et al., 2019). Therefore, we decided to add polyvinyl alcohol (PVA) to the substrate of the SNM to control the interactions between the methylol group of P(NIPAAm-co-HMAAm) and hydroxy group of PVA derived from hydrogen bonding (Cho et al., 2010; Nuruddin et al., 2021).

**SCHEME 1:**
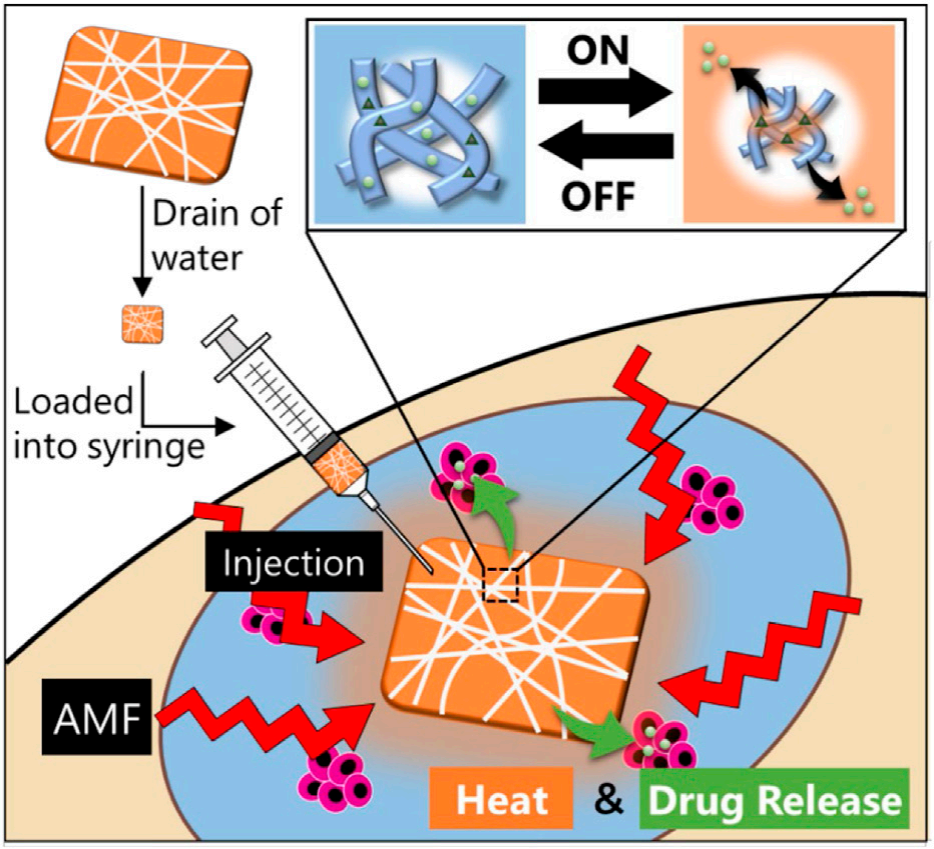
Design of a smart injectable hyperthermia nanofiber system with MNPs dispersed in temperature-responsive polymers. The nanofibers also incorporate an anticancer drug. With the device signal (AMF) turned “on” to activate the MNPs in the nanofibers, the MNPs generate heat to collapse the polymer network in the nanofiber, thus allowing “on-off” release of the drug. Following hyperthermia and chemotherapy, the heat and drug release induce apoptosis of the cancerous cells.

This study was aimed at applying the SNM to the affected area by injection without surgery. The injectable SNM not only enables minimally invasive implantation in the patient’s body but also has the advantage of improved handling performance. A minimally invasive SNM based on the P(NIPAAm-co-HMAAm) and PVA was developed for application by injection. This SNM responds to an alternating magnetic field (AMF) owing to the loaded MNPs and shows temperature-responsive drug release behavior (Scheme 1). Therefore, the well-defined multifaceted platform represents an appreciable SNM for efficient and minimally invasive distinctive therapy to the tumor cells, which is important in the validation of strategic pursuit of promoted combination therapeutic outcomes through hyperthermia and chemotherapeutics.

## Results and discussion

### Fabrication of smart nanofiber mesh

**SCHEME 2:**
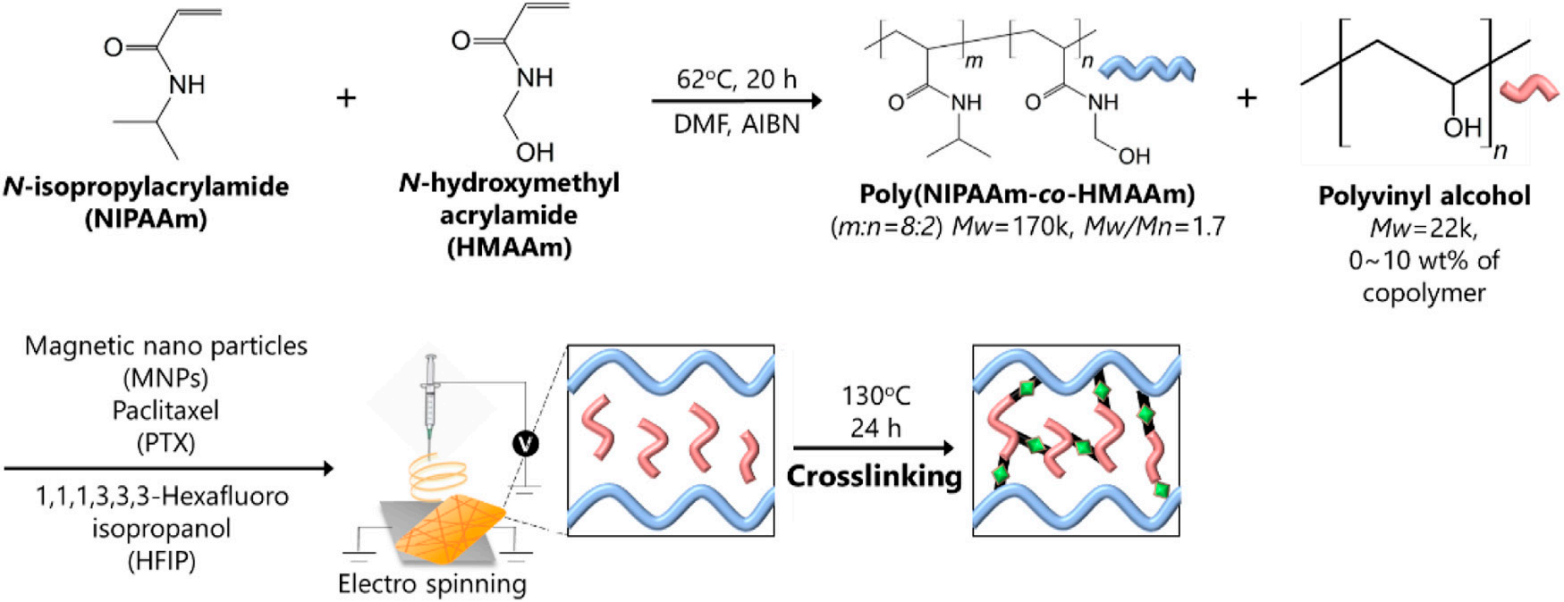
Diagrammatic illustration of the SNM with poly (NIPAAm-co-HMAAm)/PVA.

As shown in Scheme 2, NIPAAm and HMAAm were randomly copolymerized to form P (NIPAAm-co-HMAAm). In this study, a copolymer with a relatively high molecular weight (Mw 50 k∼) was synthesized. As a result of insufficient molecular chain entanglement during electrospinning, the lower molecular weight may cause beads or particles to form. As molecular weight increases, fiber morphology changes from beads to fine fibers. According to our previous report (Oroumei et al., 2015; Ahmadian et al., 2021), PNIPAAm with Mn = 10 k forms a bead-like structure at 1.0–10 w/v%. With PNIPAAm with a relatively higher molecular weight, fibers were electrospun on a lower solution concentration of 1.0–3.0%.

To adjust the shear-thinning property of SNM to achieve injectability, the hydrophilic ability will be introduced. Herein, the fiber fabrication conditions were optimized as follows: 25-gauge needle, 20 wt% polymer concentration, three different amounts (0, 5, 10 wt%) of PVA, 20 kV of applied voltage, and a 13 cm gap between the collector and the needle. A comparison of the isopropyl group of the NIPAAm and the methylol group of HMAAm by ^1^H NMR determined that the HMAAm content in the copolymer was 20 mol%. Approximately 41–43°C was selected as a mild-hyperthermia temperature for the copolymer’s phase transition. The corresponding phase transition temperature value was 42.1°C.

### Characterization of smart nanofiber mesh

**FIGURE 1:**
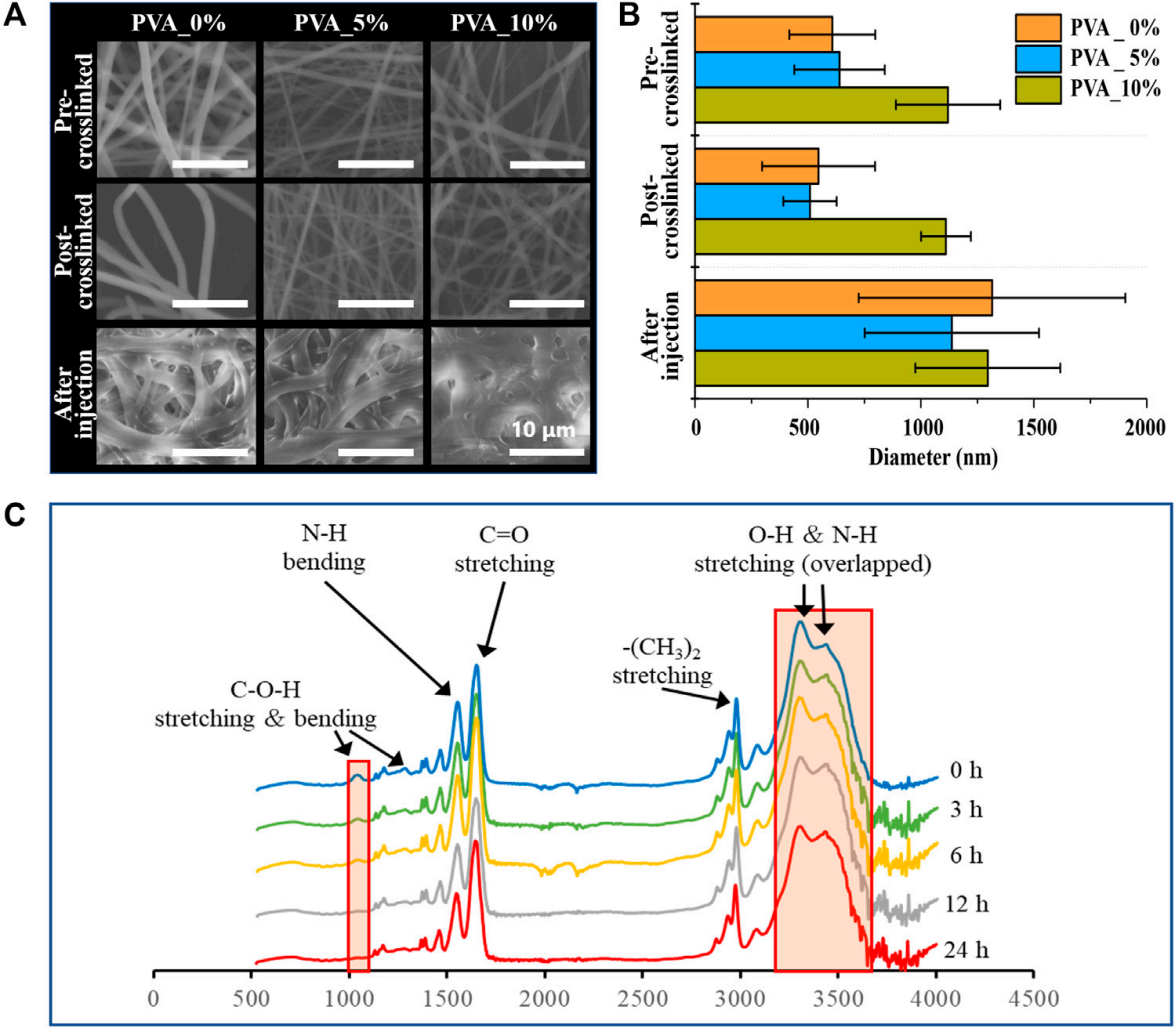
**(A)** SEM image and **(B)** the average diameters of the nanofibers: Poly (NIPAAm- c o-HMAAm) pre-crosslinked with PVA, poly (NIPAAm- c oHMAAm) post-crosslinked with PVA and poly (NIPAAm- c o-HMAAm) crosslinked with PVA after injection. **(C)** Change in 1H NMR spectra of the poly (NIPAAm- c o-HMAAm)/PVA processed with different heating times.

In this study, poly (NIPAAm-co-HMAAm)/PVA nanofibers with different amounts of PVA were fabricated through blend electrospinning. Figure 1A shows the optical microscopy and SEM images of a variety of electrospun nanofiber meshes after thermal crosslinking. As shown in Figure 1A, the nanofiber before crosslinked were bead-free, smooth and uniform fibers were formed with an average diameter of 1,120 ± 231, 640 ± 200, 608 ± 190 nm, respectively, regardless of PVA content. It is maybe the introduced of HMAAm units is to crosslink polymer chains, and this induced the stability of the meshes in aqueous medium (Kim and Matsunaga, 2017; Niiyama et al., 2018; Zha et al., 2021). Moreover, after thermal crosslinking, the nanofibers maintained nanofibrous structures and the average diameter was 1,111 ± 110, 509 ± 118, 547 ± 250 nm, respectively (Figure 1B). These results suggest that thermal crosslinking occurs only within the fibers and does not contribute to inter-fiber adhesion.

Furthermore, it has been confirmed that the nanofiber poly (NIPAAm-co-HMAAm)/PVA has a crosslinked structure by the disappearance of peaks corresponding to methylol groups in the attenuated total reflection Fourier-transform infrared spectroscopy (ATR-FTIR) spectra (Figure 1C). The peaks at 1,650 and 1,550 cm^−1^ were assigned to amide I (C=O stretching) and amide II (N–H bending) of the copolymer, respectively. The broad absorption band observed around 3,430 cm^−1^ was assigned to the N–H stretching of amide groups in the copolymer. The peaks at 1,370–1,390 and 2,980 cm^−1^ were assigned to the respective stretching modes of NIPAAm’s–CH(CH_3_)_2_ and –(CH_3_)_2_ groups. The peaks at 1,050, 1,230, and 3,300 cm^−1^ were assigned to the C–O–H stretching, C–O–H bending, and O–H stretching modes in HMAAm, respectively (Niiyama et al., 2018; Wei et al., 2022). With increasing thermal treatment time, the signal intensity of the methylol groups gradually decreased. It was confirmed that the signals completely disappeared and the thermal crosslinking was completed in 6 h.

### Shear thinning properties of smart nanofiber mesh

In recent years, electrospun nanofibers have made rapid advances in cancer therapy due to their high drug loading rates, high specific surfaces, and good drug release profiles. However, drug-loaded nanofibers are typically applied externally or injected through fibrous membranes to treat cancer, resulting in reduced complicated surgical operations, drug utilization, and secondary damage. For the purpose of solving these bottlenecks, injectable electrospun nanofibers (PNIPHM_PVA0, PNIPHM_PVA5, and PNIPHM_PVA10) thermally crosslinked with different ratios of PVA were fabricated. For investigating the injectability of the nanofiber meshes, the strain amplitude-dependent dynamic viscoelasticity, shear rate dependent viscosity, and continuous-step strain measurement were performed.

**FIGURE 2:**
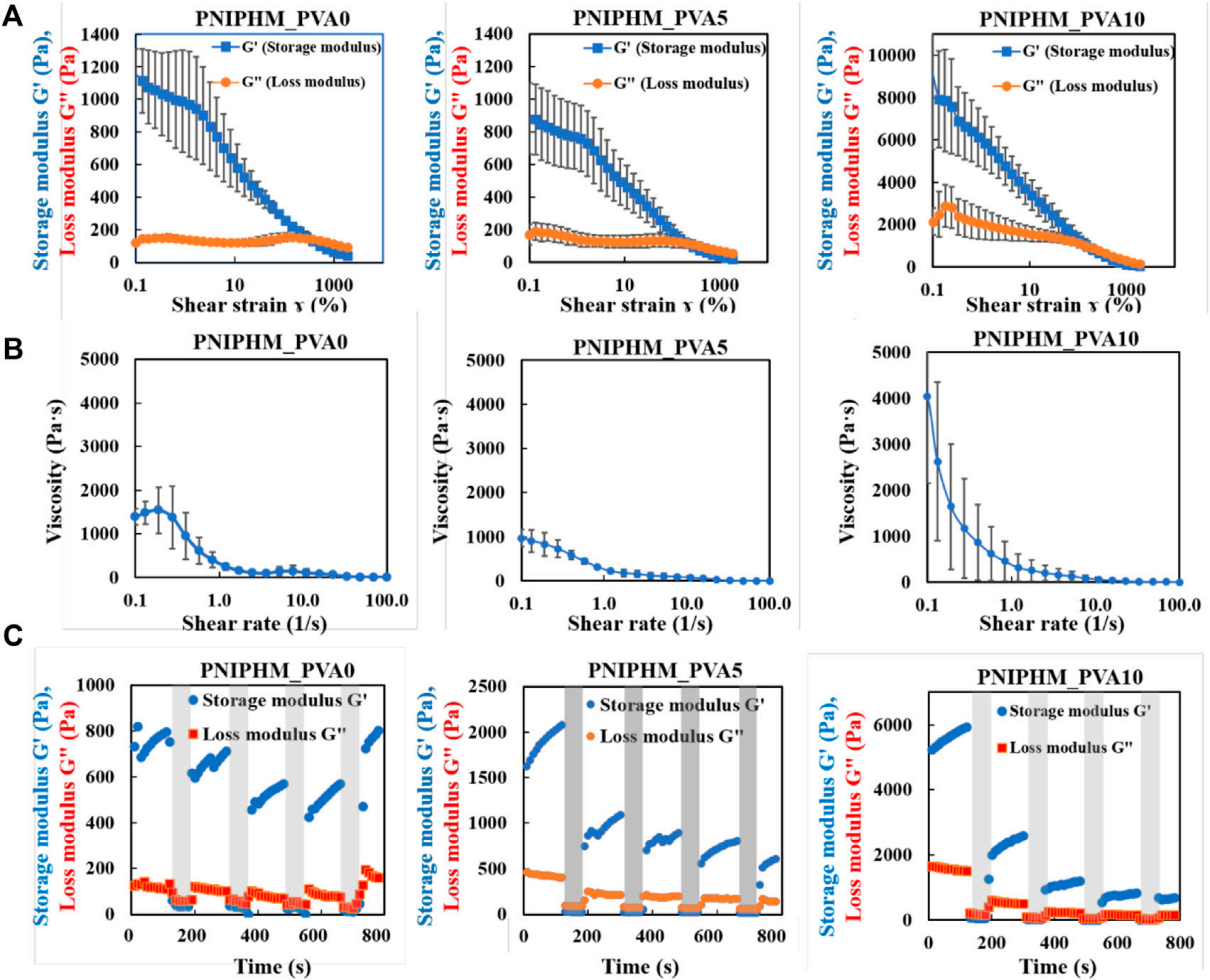
**(A)** Result of strain amplitude dependent dynamic viscoelasticity measurement, **(B)** Result of shear rate dependent viscosity measurement, and **(C)** Result of continuous-step strain measurement (*n* = 3, mean ± SD).

The loss modulus of each SNM exceeded the storage modulus when the strain is applied above a certain threshold value (Figure 2A). The strain value at the point where the loss modulus first exceeded the storage modulus was 379% for PNIPHM_PVA0, while it was 200% for PNIPHM_PVA5 and PNIPHM_PVA10. This result confirms the increase of flowability with the increase rate of PVA. Furthermore, the results of shear rate dependent viscosity measurement were shown in Figure 2B. The shear-thinning property of SNM with any PVA content decreases with an increasing shear rate. The shear-thinning property of SNMs was confirmed. In addition, it can be noted that for all types of SNM, the storage modulus was higher when the shear strain was 1% and the loss modulus was higher when the shear strain was 1,000% in Figure 2C. This indicates that the mesh becomes fluid only when shear strain is applied and recovers its shape when the strain is removed. Moreover, at 1% shear strain, the storage modulus increased with time. This indicates that the nanofiber mesh absorbs water from the surrounding area after being subjected to strong shear force, and gradually recovers its shape by swelling again. The storage modulus at the beginning of the measurement (0 s) was set to 100%, and the shape recovery rate was calculated by comparing the storage modulus at 120, 300, 480, 660, and 780 s. The shape recovery rates were 103, 97, 78, 78, 109% for PNIPHM_PVA0, 128, 67, 55, 49, 38% for PNIPHM_PVA5, and 113, 50, 23, 16, 13% for PNIPHM_PVA10. It was found that the shape recovery rate worsened with the increasing PVA addition rate. A possible explanation is that the addition of PVA, a crystalline polymer, increases the crystallinity of the nanofiber mesh, which in turn increases the mechanical strength and decreases flexibility (Faris et al., 2021; Liang et al., 2021; Zhou et al., 2022). On the basis of those results, this composition (PNIPHM_PVA5) was selected for further study.

### AMF-responsive heat generation

Thermal therapy known as magnetic hyperthermia involves heating tumors using magnetic nanoparticles and applying an AMF to create heat (Albarqi et al., 2020; Gavilan et al., 2021). High temperatures (42–45°C)can kill cancer cells, as well act as a sensitizer to enhance the effects of chemotherapy (Ahmed K, 2012). As a result of its excellent ability to penetrate tissues, AMF can precisely treat deep tumors in organs (Hayashi et al., 2016). In this regard, it is crucial to determine the heating potential of MNPs within nanofiber meshes exposed to AMF. Generally, magnetic fields affect tissues and organ systems in certain ways, such as causing induced eddy currents in tissues, resulting in necrosis or carbonization in healthy tissues. Thereby, magnetic field forces are restricted. The AMF safety frequency threshold is 100–300 kHz in clinical applications (Mamiya et al., 2017; Chen et al., 2021). In this study, an AMF of 281 kHz was used and its intensity is relevant for clinical applications.

**FIGURE 3:**
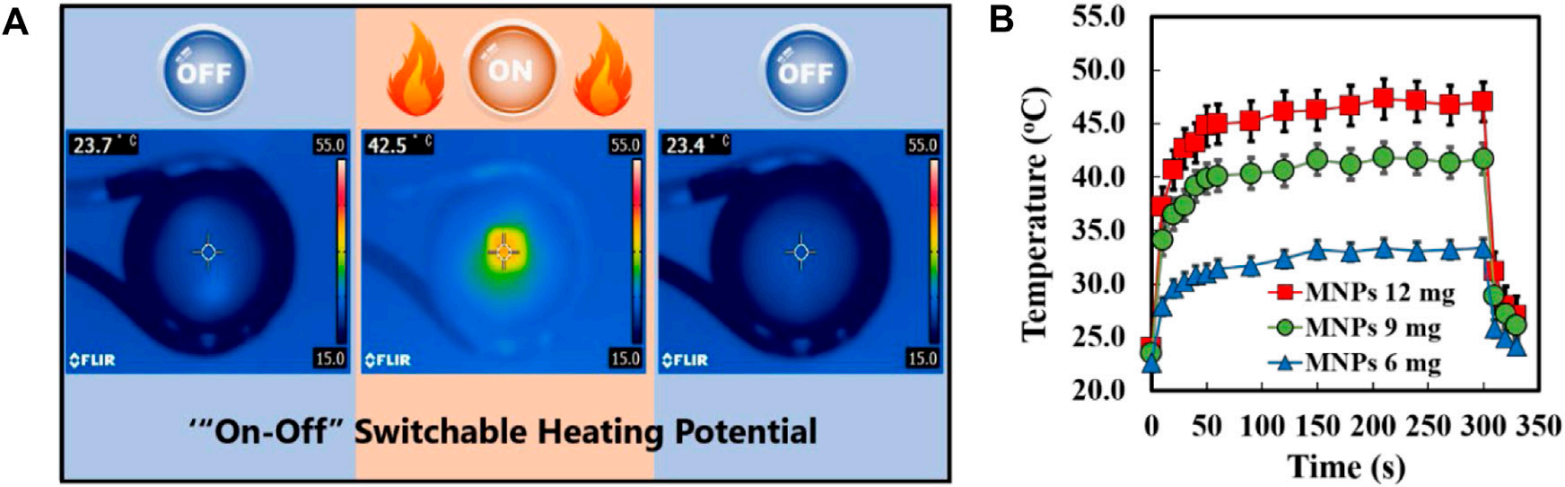
**(A)** Infrared thermal images of MNPs-loaded SNM with “on” or “off” AMF irradiation. **(B)** Heating profiles of MNPs@SNM with different MNPs contents during AMF application at different times.

To investigate the responses of the MNPs-loaded nanofibers to a magnetic field, Figure 3A shows the heating profiles of the SNMs with 30 wt% MNPs loading with AMF switching every 360 s (on for 300 s and off for 60 s). The temperature of the SNM increased from 23.7°C to 42.5°C caused by the irradiation of 281 kHz AMF and dropped immediately to the starting temperature once the irradiation was turned off. Furthermore, in Figure 3B, it shows the time-dependent temperature changes of the SNMs with different MNPs loading rates under AMF irradiation for 360 s. The temperature of each test sample sharply increased immediately after AMF irradiation, reaching a plateau within 30 s at 33.4°C, 42.1°C, and 47.3°C in meshes with 10 wt%, 20 wt%, and 30 wt% MNPs loading, respectively. The result indicated that the heating profiles are dependent on the concentration of MNPs loading, which will make the heating ability of the SNM more controllable. Based on these results, the SNM with 30% (9 mg) of MNPs was chosen for further investigation.

### “On-off” switchable drug release

**FIGURE 4:**
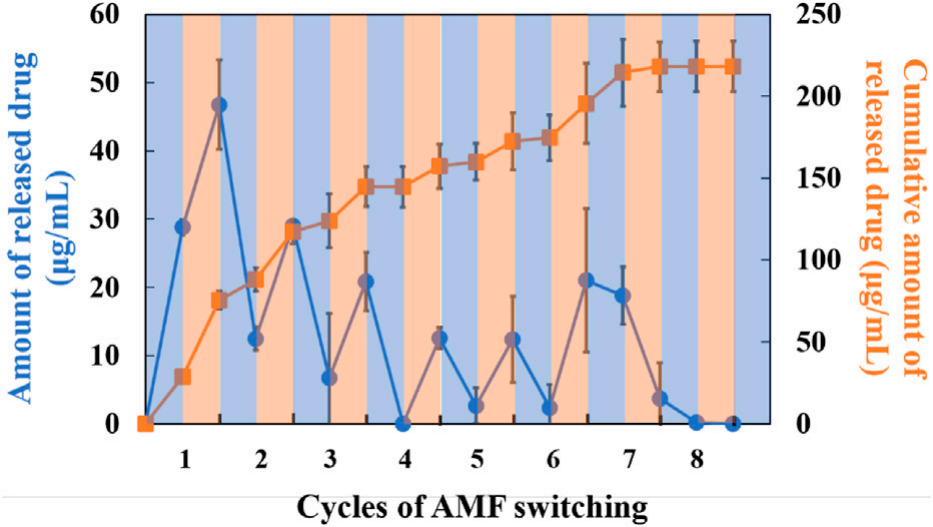
Drug release behavior from SNM containing PTX (1.0 wt%), MNPs (0 or 20 wt%) and PVA (5 wt%) at different cycles of AFM.

Controllable drug release is an important evaluation criterion for SNM. Next, the nanofiber meshes containing PTX, MNPs and PVA were used to verify the drug release behavior from nanofiber. NIPAAm-co-HMAAm polymer is soluble in aqueous media below LCST and dehydrates rapidly above LCST. Therefore, PTX is released from the polymer along with the heating of MNPs induced by AMF. Figure 4 shows the switching from “on” to “off” AMF applications on PTX release from nanofibers. It is worth noting that approximately 16% of loaded PTX were released in the first heating process. However, the release was stopped after cooling to room temperature, and restarted release during the second heating. It is demonstrated that the release of the PTX was accelerated by AMF irradiation and was stopped at room temperature. Interestingly, with the increase of the switching cycle number, the cumulative release of PTX also increased significantly, and showed a switching cycle-dependent relationship. The release of the PTX cumulative amount was over 70% within eight cycles. Owing to the high specific surface area of nanofibers, their sensitivity to external stimuli is greater than that of bulk materials (Lin et al., 2013). Thus, we took advantage of the nanometric effects of nanofibers for the “on-off” switchable release of drugs. From this regard, these results in Figure 4 indicate that the proposed SNM system allows the control of switchable drug release by simply switching the AMF “on” and “off,” and this controlled release SNM may be very promising for cancer therapeutics in the future.

### Anti-tumor effect

Hybrid nanoarchitectures with magnetic nanoparticles have been used for hyperthermic cancer cell therapy in recent years (Lin et al., 2013). However, due to their nano dimensional properties, these hybrids cannot be directly applied to living matter, as they may lead to toxic side effects. Interestingly, one of the advantages of the SNM in this study is that a nanofiber as a dressing can be manipulated and injected directly into the tumor region. In this regard, the anti-tumor effects of the SNMs based on hyperthermia and the chemotherapeutic effects of the MNPs/PTX@SNM was investigated using SKOV3 cells.

**FIGURE 5:**
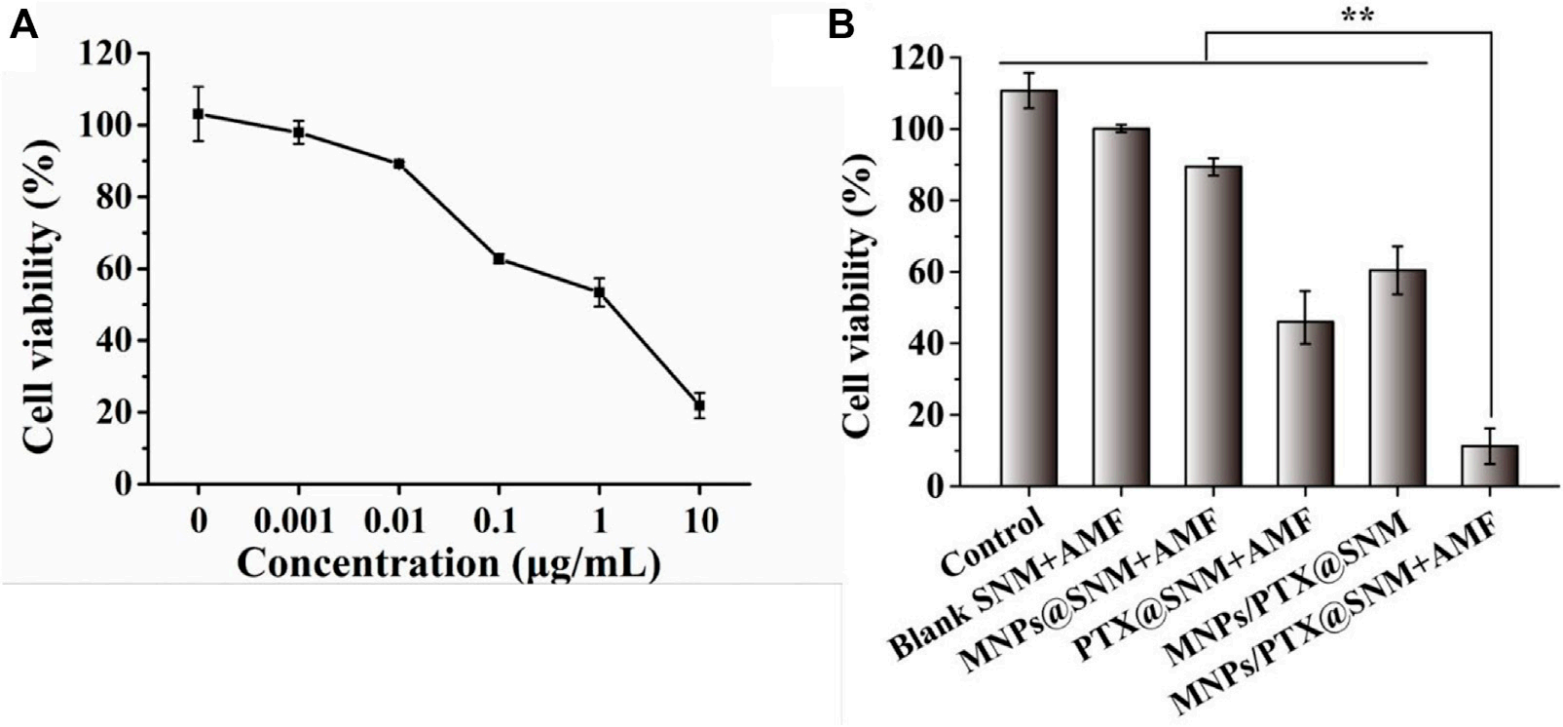
Cell viability of SKOV3 cells treated with **(A)** different concentrations of PTX, and **(B)** nanofiber meshes with different cycles of AFM. (Data are mean ± SD *n* = 6, **p < 0.01).

Before co-culturing with nanofiber mesh the cell viability of SKOV3 cells treated with PTX was assessed by the alamar Blue assay. A culture medium containing PTX was used to culture the SKOV3 cells at 0, 0.001, 0.1, 1.0, and 10 μg ml^−1^ concentration for 24 h. As shown in Figure 5A, cell viability decreases as the drug concentration increases, and IC_50_ of SKOV3 at 1.66 μg ml^−1^. Furthermore, the anti-tumor effects of the SNMs were further evaluated using SKOV3 cells. The cells were co-cultured with the SNMs for 24 h And the cells were exposed to AMF once every 5 min and then at room temperature for 5 min for each cycle. As demonstrated in Figure 5B, the blank SNM under the AMF maintained cell viability higher than 95%, indicating that the blank nanofiber mesh and magnetic field was non-toxic against SKOV3 cells. On the other hand, with the SNM exposure to AMF, the cell viability of SNM loaded with MNPs alone (MNPs-SNM) and PTX alone (PTX@SNM) decreased gradually compared to the control group (medium only). This may be due to the effect of hyperthermia generated by MNPs-loaded SNM exposed to AMF and chemotherapy effect resulting from PTX release from surface of nanofiber. Moreover, the cells viability in the presence of MNPs and PTX co-loaded nanofibers group was 60.45 ± 6.72. This result may be due to few PTX being released from the surface of nanofibers without AMF application (“off” state). This result corresponds well to the *in vitro* drug release study (Figure 4). It can be noted that, the cell viability of MNPs/PTX@SNM with the magnetic field was only 11.23% ± 5.01, because of the combined effect of hyperthermia with PTX released caused by heating. In sum, the findings demonstrate that MNPs and PTX@SNM exposed to AMF greatly increase cytotoxicity in SKOV3 cells. In light of this, this platform may be developed into an implantable delivery system for treating ovarian cancer.

## Experimental

### Materials

Dimethylformamide (DMF), 2′2-Azobis (isobutyronitrile) (AIBN) and *N*-Isopropylacrylamide (NIPAAm) were purchased from Fujifilm Wako Pure Chemical Corporation (Osaka, Japan). The following products were purchased from Tokyo Chemical Industry Co., Ltd. (Tokyo, Japan): *N*-Hydroxymethylacrylamide (HMAAm), 1,1,1,3,3,3-Hexafluoro-2-propanol, and Paclitaxel (PTX). We obtained ferrofluid made of iron oxide (III) (10 nm particle size) from Ferrotec Holdings Corporation (Tokyo, Japan). Fetal bovine serum (FBS) was purchased from Tocris Bioscience Inc. (Minneapolis, MN, United States). McCoy’s 5 A Medium Modified, content sodium bicarbonate, free of L-glutamine, liquid, and sterile-filtered for cell culture was purchased from Sigma-Aldrich Japan (Tokyo, Japan). We obtained the Alamar Blue reagent from TREK Diagnostic Systems (Cleveland, OH, United States). The American Type Culture Collection (Manassas, VA, United States) provided SKOV3 (human ovarian cancer cell line: adenocarcinoma).

### Synthesis and characterization of P(NIPAAm-co-HMAAm)

According to the previous description, NIPAAm and HMAAm were copolymerized. Briefly, 80 mol% NIPAAm was dissolved in 20 ml of DMF, followed by 20 mol% HMAAm and 0.01 mol% AIBN. In total, the monomers possessed a molar concentration of 50 mmol. For the copolymerization, 62°C was maintained for 20 h before four freeze-thaw cycles degassed it completely. After polymerization, AIBN, unreacted monomers, impurities, and solvent were removed by dialysis against ethanol (FUJIFILM Wako Pure Chemical Corporation, Osaka, Japan) and distilled water for 7 days. It takes 3 days for the dialyzed solutions to be lyophilized after they have been dialyzed. The chemical structure of the copolymer was verified by NMR (JEOL, Tokyo, Japan). The average molecular weight (*M*n) and polydispersity index (PDI) of the copolymer was determined by gel permeation chromatography (GPC, JASCO International, Tokyo, Japan) using DMF with lithium bromide (LiBr, 10 mm) (Tosho Corporation, Tokyo, Japan) as an eluent sample. A UV-Visible spectrophotometer (JASCO Corporation, Tokyo, Japan) with a heating rate of 1.0 C/min was used to measure temperature-dependent changes in the transmittance of the copolymer in phosphate-buffered saline (PBS) (pH = 7.4, 0.1% w/v). We defined the lower critical solution temperature of the copolymer as the temperature at which 50% of the transmission was achieved.

### Fabrication of fiber meshes

A solution for electrospinning was prepared by dissolving poly (NIPAAm-co-HMAAm) in HFIP (20 w/v%). The electrospun solution contained 30 w/w%, 1.0 w/w%, 0–20 w/w% of MNPs, PTX, and polyvinyl alcohol, respectively. The solution was electrospun into fibers using an applied voltage of 20 kV with a flow rate set to 1.0 ml/h, and 13 cm separation of the needle (25 gauge) and collector plate at 25°C and 42% humidity (Nanon-01A, MECC Co., Ltd, Fukuoka, Japan). In order to remove organic solvents from electrospun fibers, they were placed in an oven (Tokyo Rikakikai Co., Ltd, Tokyo, Japan) at 140°C for 24 h. This process involved the methylal group in HMAAm and the hydroxy group in PVA were crosslinked in the nanofiber mesh. The morphology of fibers was observed using a scanning electron microscope (SEM) (SU8000, Hitachi High-Technologies Corporation, Tokyo, Japan) with secondary electrons (SE) after Pt coating of the fiber surface. The diameter of fibers was calculated from a SEM image using ImageJ software. Crosslinking between methylal groups of HMAAm within the fiber was confirmed based on the disappearance of absorbance at 1,050 cm^−1^ through ATR-FTIR spectroscopy (IRPrestige-21, Shimadzu, Kyoto, Japan). Analysis was conducted after the residual solvent was removed from fibers by vacuum drying (ULVAC KIKO, Inc, Miyazaki, Japan).

### Rheological characterization of smart nanofiber mesh

The rheological properties were tested using a rheometer (MCR301, Anton Paar Japan K.K, Tokyo, Japan). Strain amplitude-dependent dynamic viscoelasticity, shear-rate-dependent viscosity, and continuous-step strain measurement were performed. In each measurement, the prepared SNM was swollen with distilled water and then cut into 1 cm diameter circles for measurement. In the strain amplitude-dependent dynamic viscoelasticity measurement, the angular frequency was fixed at 10 rad/s, the shear strain was varied logarithmically from 0.01% to 2000%, and the storage modulus G’ (Pa) and loss modulus G” (Pa) were measured. The number of measurement points was 32, and the total measurement time was set to 96 s. In the shear-rate-dependent viscosity measurement, shear stress (Pa) was measured by varying the shear rate logarithmically from 0.001 s^−1^ to 100 s^−1^. The number of measuring points was 26 and the total measurement time was set to 143 s. In the continuous-step strain measurement, the angular frequency was fixed at 10 rad/s. The shear strain was repeated for 120 s at 1% and for 60 s at 1,000% for a total of four cycles, and the storage modulus G’ (Pa) and loss modulus G” (Pa) were measured. The total number of measurement points was 72, and the total measurement time was 720 s.

### Nanofiber smart mesh heating profiles

MNPs within the nanofiber mesh were studied by applying AMF to determine their heat-generating properties. SNMs (6.0, 9.0, 12.0 mg) of MNPs were placed in the center of HOTSHOT2 (Alonics Co., Ltd, Tokyo, Japan) of a customized copper coil that generated AMF (480 A, 281 kHz frequency, 362 W). We measured the heating profiles at 30 and 60 s using an FL-IR thermo-camera (CPA-E6, FLIR Systems Japan K.K, Tokyo, Japan).

### Drug release from smart nanofiber mesh

In 1.0 ml of phosphate-buffered saline (PBS) at room temperature, samples of SNM (30 mg) thermo crosslinked with 5% PVA were first swollen for 5 min by shaking (at 100 rpm) (Lin et al., 2019). As soon as the sample reached equilibrium, it was placed in the center of a copper coil. Afterward, 0.25 ml of the supernatant was collected, and fresh PBS was added to the supernatant that had been irradiated with AMF or cooled for 5 min at room temperature.

PTX release profiles were quantified by UV-visible spectroscopy during each switching cycle. (V-650 spectrophotometer, Jasco, Tokyo. Japan). Eight cycles were repeated. A cumulative release of PTX was calculated using the following equation: $W_{n}=W_{n-1}+\left\{C_{n}-C_{n-1}\left(1-l\right)\right\}$ , where *W*_n_ (μg) and *C*_n_ (μg ml-1) are the cumulative release amount and concentration of PTX at the n th (*n* = 1–16) collection process, respectively, and *l* is the volume collected PBS (=0.25 ml).

### Cell preparation

The cells used in this study were SKOV3 (human ovarian adenocarcinoma). McCoy’s 5 A medium was used as a growth medium with 10% fetal bovine serum and 1% penicillin-streptomycin. To prepare SKOV3 cells, subcultures in a tissue culture dish (100 mm) were incubated for 2 days at 37°C in 5% CO_2_ (MCO-170AICUVH, PHC Holdings, Tokyo, Japan). The following experiments collected the cells from a confluent monolayer in the dish with 0.25% (w/v) trypsin and resuspended them in 10 ml of growth medium.

### Drug sensitivity of SKOV3 cells

Approximately 1.0 × 10^5^ SKOV3 cells were seeded on a 96-well plate, 200 µL of the growth medium was added and incubated for 24 h. The medium was exchanged for 96-well plates which contained 0, 0.001, 0.01, 0.1, 1.0, and 10 μg ml^−1^ of PTX, and incubated for 24 h. After adding Alamar Blue (ten percent of the medium), each well was incubated at 37°C for 4 hours. The number of cells was calculated by measuring the fluorescent intensity at 570 nm excitation and 600 nm emission (Tecan Japan). Cell viability and IC_50_ were calculated based on considering the number of cells in the control group (without PTX treatment) as 100% viability.

### AMF-responsive anti-tumor activity *In vitro*

For the anticancer experiment of the SNM, SKOV3 cells were seeded in a 6 mm plate at 2 × 10^6^ cells per well for 24 h. Thereafter, the cells incubated a piece of nanofiber mesh cross-linked with 5% of PVA (which had a 30 mg weight, 30% MNPs and 1% PTX), which was exposed to AMF irradiation (480 A, amplitude 281 kHz frequency) for 5 min. After further incubation at 37°C for another 24 h, the Alamar Blue assay reagent (10% against medium) was added to each well and incubated for 4 hours at 37°C, according to protocol. The cell number was computed from the fluorescent intensity measured by a fluorescence plate drive set at 570 nm excitation and 600 nm emission.

### Statistical analysis

All experiments were conducted three times and the data are presented as means ± standard deviation (SD). Statistical analysis was performed using the student t-test and variance one-way analysis (ANOVA) by Origin version 9.0 (Northampton, United States). The difference between the results was considered to be statistically significant for *p* < 0.01 (**).

## Conclusion

In the current research, switchable drug release injectable nanofiber platforms were engineered for simultaneous for chemotherapeutic and hyperthermia to cancerous cells. The SNM were fabricated by an electrospinning method with a temperature-responsive polymer, MNPs, and PTX, and the fiber was further cross-linked with PVA. Nanofibers responded to alternating “on”, and “off” switches of AMF application and the controllable release of the drug from the fibers was observed as a result. Aiming for amplification of chemotherapeutic potency, MNPs-loaded SNM schemed. The SNM exposure to AMF enables switchable drug release and generates hyperthermia effects, which causes heat-induced cell killing as well as enhanced PTX chemotherapeutic efficiency. Subsequent investigations approved the validity of our strategic hyperthermia in amplifying chemotherapeutic potency; namely, the SNM efficiently induced apoptosis of SKOV3 cells through the synergistic anticancer effect arising from hyperthermia and PTX. Hence, the current research not only supported an intelligent nanofiber platform for injectable and controllable drug release to be fabricated, but also urged the promising potential of exploiting combination strategy in advancing tumor therapy.

## Data Availability

The original contributions presented in the study are included in the article/Supplementary Material, further inquiries can be directed to the corresponding author.
